# Voltage-Gated Ion Channels in Cancer Cell Proliferation

**DOI:** 10.3390/cancers7020813

**Published:** 2015-05-22

**Authors:** Vidhya R. Rao, Mathew Perez-Neut, Simon Kaja, Saverio Gentile

**Affiliations:** 1 Department of Molecular Pharmacology and Therapeutics, Loyola University Chicago 2160 S. 1st Ave, Maywood, IL 60153, USA; E-Mails: vrao2@luc.edu (V.R.R.); mperezneut@luc.edu (M.P.-N.); 2 Department of Ophthalmology and Vision Research Center, School of Medicine, University of Missouri-Kansas City, 2411 Holmes St., Kansas City, MO 64108, USA; E-Mail: kajas@umkc.edu

**Keywords:** cancer, cell proliferation, voltage gated ion channel, membrane potential

## Abstract

Changes of the electrical charges across the surface cell membrane are absolutely necessary to maintain cellular homeostasis in physiological as well as in pathological conditions. The opening of ion channels alter the charge distribution across the surface membrane as they allow the diffusion of ions such as K^+^, Ca^++^, Cl^−^, Na^+^. Traditionally, voltage-gated ion channels (VGIC) are known to play fundamental roles in controlling rapid bioelectrical signaling including action potential and/or contraction. However, several investigations have revealed that these classes of proteins can also contribute significantly to cell mitotic biochemical signaling, cell cycle progression, as well as cell volume regulation. All these functions are critically important for cancer cell proliferation. Interestingly, a variety of distinct VGICs are expressed in different cancer cell types, including metastasis but not in the tissues from which these tumors were generated. Given the increasing evidence suggesting that VGIC play a major role in cancer cell biology, in this review we discuss the role of distinct VGIC in cancer cell proliferation and possible therapeutic potential of VIGC pharmacological manipulation.

## 1. Introduction

Cancers continue to be a leading cause of mortality and morbidity worldwide [1]. While extensive research over the years has resulted in better diagnosis and treatment options, there still is a significant dearth of effective, affordable and safe therapeutics that can improve the prognosis and the life style of affected patients [2,3]. A vast number of studies have significantly contributed to the current understanding of molecular mechanisms contributing to cancers which ranges from specific genetic mutations, to intricate biochemical and molecular pathways [4,5]. Albeit the complexity and heterogeneity of the disease, uncontrolled cell proliferation of individual cancer cells is essential for initiation and further progression of the disease [6,7]. More recently, ion channels have been recognized to play an important role in cancer pathology as they are capable of regulating key events in cell proliferation and therefore, cancer initiation and progression [8,9,10]. Interestingly, aberrant expression of specific ion channels and activity has been associated with various stages of cancer and increased aggressiveness. Furthermore, *in vitro* and preclinical studies have revealed that pharmacological manipulation of channel activity offers protection against several cancers. Ion channels therefore offer a novel strategy that can be potentially utilized to treat cancers. In this article, we review the unique role of a specific class of ion channels, the voltage-gated ion channels, in regulating cell proliferation and therefore their contribution to development and progression of cancer.

### Membrane Potential and Cell Proliferation

**Figure 1:**
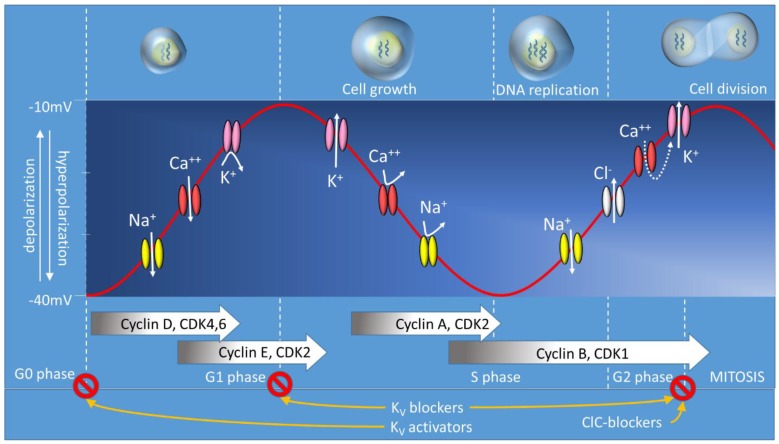
Schematic representation of possible involvement of different VGIC activity during the cell cycle of cancer cells. According to their expression level, several VGIC have been found playing important roles during the cell cycle. Opening of the voltage-gated Na^+^ and/or Ca^++^ channel move positive charges from the extracellular space to the cytoplasm causing depolarization of the membrane. This event appears to be essential to promote transition from the G0/G1 phase to the S phase of the cell cycle. In contrast, membrane potential during the S phase tends to repolarize due to the opening of K^+^ channels and/or the closing of Ca^++^ and/or Na^+^ channels. Mitosis is associated with more depolarized membrane potential compared to cells in the rest of the S phase. This is possible due to increased cytosolic Na^+^ and/or Ca^++^. Depolarization and augmented Ca^++^ entry will allow stimulation of Ca^++^-activated K^+^ channels and activation of Cl^−^ channels. Both K^+^ and Cl^−^ outward currents are responsible for water leaving the cytoplasm, which leads to a cell shrinkage before cell division. Chronic application of K^+^ channel blockers (e.g., Kv11.1 blocker E4031) or openers (Kv11.1 opener NS1634) leads to changes of the membrane potential in the opposite direction, but both type of drugs cause arrests of the cell cycle in the G0/G1 phase, while blockade of the VGClC arrests the cell cycle in the G2 phase. This suggests that oscillation of the membrane potential is a fundamental event that promotes progression of the cell cycle. ↓ = inward ionic flux; ↑ = outward ionic flux; 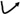 = no ionic flux.

Cell proliferation in normal cells is a complex, well synchronized event that is stringently regulated by a number of ions, molecules and proteins associated with the cell cycle machinery including Ca^++^, ATP, cyclins, cyclin dependent kinases and many other cell cycle regulators [11,12]. A cell cycle can be distinguished into phases (Figure 1), namely, the G0 phase, comprised mainly of the non-proliferating cells, G1 phase with cells getting primed for DNA replication, followed by the S phase with cells undergoing DNA replication, leading to the G2 phase, where the cells are getting ready to undergo mitosis/cell division. Finally, the mitosis (M) phase results in complete division of the cells with daughter cells ensuing that individually continue the process of cell cycle [11,12]. One of the most significant and dynamic factors that regulates cell cycle is the membrane potential (Vm; Voltage membrane) [13,14]. Vm (also called transmembrane potential) is an electrical charge that is created by the discrepancy in ionic concentration between the intracellular and extracellular environment. Ion channels and ion transporters play a fundamental role in generating Vm as they are selectively permeant to ions that can cross the membrane according to chemical and/or electrical gradient. As a result of their activity, the Vm of a resting cell is negative. The cells are said to be depolarized (Figure 1) when the Vm is altered to relatively less negative state, whereas the cells are said to be hyperpolarized, when the membrane potential is moved to more negative values than the resting membrane potential [15]. A number of studies have reported that cells with a much hyperpolarized resting potential, such as muscle cells and neurons, typically show little or no mitotic activity, while proliferating cells, particularly cancer cells, have a depolarized membrane potential in comparison to normal cells [11,13,15,16,17,18]. In the seminal studies conducted in sarcoma cells by Clarence D. Cone Jr., it was observed that Vm underwent a transient hyperpolarization before entering mitosis, followed by a rapid depolarization through the M Phase suggesting that Vm varies through the cell cycle progression [2]. Further, it was observed that lowering the Vm to a hyperpolarized state similar to that of neurons, contributed to a mitotic block in proliferating CHO cells, while a sustained depolarization could induce DNA synthesis and mitosis in mature neurons [16,17,18]. In MCF-7, a breast cancer cell line, it has been observed that the Vm during a cell cycle progression correlates with the transition in each phase, such that, the pharmacological arrest of MCF-7 cells in G1/S or G2/M transition enriches cells with hyperpolarized Vm while cells arrested in the G0/G1 and M Phases had enriched cells with depolarized Vm. [19]. Similarly, in neuroblastoma cell lines, cell cycle progression was observed to correlate with hyperpolarized Vm in G1-S transition and depolarized Vm at the M phase [20]. Thus, progression of the cell cycle is accompanied by rhythmic oscillation of the Vm accompanied by transient hyperpolarization and depolarization [14,18,20,21,22,23,24]. Voltage-gated ion channels (VGICs) are a distinct group of ion channels that are selectively permeable to Na^+^, K^+^, Ca^++^ or Cl^−^ and respond to changes in the membrane potential [25,26,27]. Besides their classical role in excitable cells, which include generating action potentials in neurons or contraction in muscles, voltage-gated ion channels also play vital roles in non-excitable cells including maintenance of cellular homeostasis by controlling ion transport, fluids, volume regulation and as well as proliferation.

## 2. Voltage-Gated Potassium Channels

Voltage-gated K^+^ channels (VGKC; Kv), as the name suggests, are selectively permeable to K^+^ ions and comprised of a large family of heterogeneous groups of ion channels forming 12 subfamilies (Kv1–Kv12). Activities of these proteins are regulated by the changes in the membrane potential and are critical for governing the resting membrane potential in both excitable and non-excitable cells [28]. Several VGKC have been found aberrantly expressed in different cancer tissues but not in the tissues from which they generated. In addition, the alteration of VGKC activity exhibited a strong impact on several aspects of cancer biology ranging from the arrest of cell proliferation to the inhibition of motility. In what follows, we discuss the roles of several VGKC that have been involved in cancer.

### 2.1. The EAG Superfamily of Potassium Channels in Cancer Biology

The voltage sensitive ether à-go-go potassium (EAG 1) channel, also referred to as Kv10.1, encoded by *KCNH1* gene has been particularly investigated for its role in cancer cell proliferation. The normal expression of Kv10.1 is limited to the brain; however, aberrant expression of this channel has been reported in a number of tumors and cancer cell lines, and it has been associated with poor prognosis [29,30,31,32,33,34,35]. Although the mechanism linking Kv10.1 activity to proliferation is still not clear, it appears clear that Kv10.1 plays a fundamental role in cancer biology. This conclusion is centered mainly on data showing that the inhibition of Kv10.1 current activity not only reduces the proliferation of cancer cells and tumors in *in vitro* and *in vivo* models [36,37,38,39], but also overexpression of this channel alone is able to increase cell proliferation and render the cells tumorigenic [29]. More recently, EAG2 (Kv10.2), an isoform of Kv10.1 has been reported to play a key role in a medulloblastoma progression. A knock-down of Kv10.2 results in tumor inhibition in mouse models of medulloblastoma and the tumor suppression has been attributed to inhibition of mitosis via cell volume regulation.

The human ether à go-go-related gene 1 (hERG1/*KCNH2*) encodes for the Kv11.1 channel that is expressed in several organs in normal adults, including in heart, brain and hematopoietic cells [40,41,42]. Interestingly, it has been observed that, expression Kv11.1 is selectively upregulated in a variety of cancers [43] and several studies have shown that Kv11.1 channel can be a potential novel biomarker for cancer invasion and survival. Inhibition of Kv11.1 results in reduced proliferation of cancer cell lines and activation of an apoptotic event resulting in cell death [44,45,46,47].

Interestingly, changes in protein levels of cyclin E2 and p21^Waf1/Cip1^ are Ca^++^ dependent [48]. Therefore, changes of Kv channel current activity can alter Ca^++^ homeostasis which can result in activation or inactivation of signaling that is critical for cell proliferation [49].

Interestingly, Kv-related oncogenicity is not necessarily only associated with ionic conduction as it has been shown that the voltage-dependent gating of EAG can control the Mitogen Activated Protein Kinase signaling pathways (MAP kinase) and cell proliferation by a mechanism that is independent from K^+^ flux through the channel [50]. A possible explanation for this event is that the morphological changes occurring to the channel during gating might expose a specific domain that serves as a docking/activator of the MAP kinase signaling. Therefore, the channel exhibits also functions of scaffolding. Moreover, it was observed that, a mutation in EAG1 that eliminates ion permeation failed to abolish xenograft tumor formation and vascularization by transfected cells possibly via increasing HIF—1apla activity [51]. Similarly, non-conducting mutants of Kv1.3 channels retained their pro-proliferative effects on vascular smooth muscle cells [52]. These provide an interesting example of how ion channels can play important roles in normal and/or cancer cell biology independently from their ability to transport ions across the membranes.

The increase in K channel activity corresponding to a transient hyperpolarization observed during the cell cycle seems to be essential for cell cycle progression and proliferation [53,54,55]. The transient hyperpolarization could serve as a potential drive from passive Ca^+^ influx, which is essential for initiating cell proliferation [56,57,58]. However, the aberrant or selective increase in Kv channels in tumors or cancer cell lines is poorly understood.

The increase in expression and activity of Kv channels in cancer cells can be attributed to genetic factors, as in the case of genomic amplification observed for Kv10.1 resulting in its overexpression in some cancers [35,59]. Epigenetic mechanisms including DNA methylation and histone acetylation has been implicated in the altered expression of several ion channels [60,61,62]. The altered expression of Kv channels could also be attributed to tumorigenic mechanisms including, for example, mutations in p53 gene and a number of oncogenic stimuli such as epidermal growth factor [63].

Interestingly, altered Kv channel activities can also be related to steroid hormone-activated signaling that does not involve transcription of the channel. For example, several groups have reported that Kv11.1 channel activity can be modulated by a non-genomic steroid hormone receptor-mediated mechanism [64,65,66,67,68] suggesting that Kv11.1 can play a major role in hormone-driven cellular response in cancer biology.

Given the changes in the membrane potential during cell cycle progression, the possible role of Kv channels in regulating cell cycle and proliferation has been extensively investigated. Interestingly, the expression and activity of certain Kv channels seem to be regulated differentially through the course of cell cycles both in normal and cancer cells. For example, increase in Kv channel density including that of Kv1.3, Kv10.1 (EAG1), Kv10.2 or Kv11.1 (HERG) has been observed to increase through M and G1 contributing a transient repolarization and a subsequent decrease in activity during the S and G2 phases in various cell lines [69,70,71,72,73].

It has been well established that ion channels work in concert as the activity of one type of channel can determine dramatic changes of ionic flux of another ion channel. This phenomenon occurs independently from the cell type and suggests that ion channels can self-regulate their function according to the cellular need. For example, neurons can accommodate overexpression of a specific voltage-gated member of the shaker family of ion channel by inducing expression of a member of another ion channel family such as the HCN channel [74]. This event appears to be independent from the shaker channel current activity and results in activating a compensatory mechanism that allow cells to preserve homeostasis. Therefore, oncogenic properties of ion channels are observed or amplified in cells that do not have the necessary tools to compensate overexpression of a specific ion channel.

### 2.2. Other Kv Channels

Kv1.3 belongs to the shaker family of voltage-gated K^+^ ion channels. Kv1.3 is known to function in conjunction with KCa3.1, a Ca^++^ activated potassium channel in physiologic lymphocyte activation and proliferation. However, expression of Kv1.3 channel in non-excitable cells revealed a strong inhibition of protein tyrosine kinases suggesting that changes in membrane potential can affect kinase-dependent signaling [75]. An increase in activity of both Kv1.3 and KCa3.1 has been implicated in several cancer types and inhibition of these channels also inhibits cell proliferation [76]. Mitochondrial Kv1.3 have been reported to play important roles in cancer cell apoptosis [77]. Inhibition of mitochondrial Kv1.3 using cell permeant inhibitors mediates apoptosis and inhibits tumor growth *in vivo*. The apoptosis mediated by my Kv1.3 inhibitors seems to be mediated via both extrinsic or intrinsic pathways suggesting that Kv1.3 inhibition could be helpful in treating cancers with deregulated apoptotic signaling pathways [78,79]. Similarly, it has been reported that mitochondrial Kv1.5 activity and expression is suppressed in various cancer cell lines including, glioblastoma, breast cancer cell lines, and bringing the levels of Kv1.5 back to normalcy results in cell growth inhibition via [80] apoptosis.

The Big Potassium (BK) channel encoded by the *KCNMA1* respond to changes in membrane potential and/or increased intracellular calcium. BK has been found abundantly expressed in meningiomas and gliomas in which they presented a higher sensitivity to calcium [81,82,83]. This suggests that this channel can contribute to cell proliferation by responding promptly to the small changes of intracellular calcium during cell cycle.

## 3. Voltage-Gated Calcium Channels

Voltage-gated Ca^++^ (Ca_V_) channels are critical mediators of Ca^++^ influx in excitable cells, where they are responsible for the control of neurotransmitter and hormone secretion, gene expression, muscle contraction, and many other physiological processes [84,85]. Gain of function mutations associated with neurological diseases and pathologies can lead to excessive activation of Ca_V_ channels, resulting in acute or chronic states of excitotoxicity [84]. In non-excitable cells, Ca_V_ channels have been linked to modulate and control cell proliferation and attracted interest as potential targets for anti-cancer therapy [86].

Ca_V_ channels belong to the superfamily of voltage-gated ion channels and share significant homology with both voltage-gated K^+^ and Na^+^ channels. Ca_V_ channels are classified as either high voltage-activated or low voltage-activated channels, and open upon membrane depolarization to mediate inward Ca^++^ influx [84,85]. The group of high voltage-activated channels is comprised of dihydropyridine-sensitive Ca_V_1 (L-type) channels and three subtypes of Ca_V_2 (P/Q-, N^−^ and R-type) channels, while Ca_V_3 (T-type) channels constitute the group of low voltage-activated Ca_V_ channels. Functional diversity of Ca_V_ channels is conferred both by the subtype α_1_ subunit and by extensive alternative splicing.

Both Ca_V_1 and Ca_V_3 channels have been implicated in the proliferative pathophysiology of various cancers, and their contributions are reviewed below.

### 3.1. Ca_V_1 Channels

Most evidence for the involvement of Ca_V_1 channels stems from studies on adrenal aldosterone-producing adenomas (APAs) and prostate cancers. Specifically, mutations in the *CACNA1D* gene encoding the Ca_V_1.3 channel were recently identified in APAs [87]. The mutations (Gly403 and Ile770) caused a gain-of-function phenotype at the single channel level, resulting in activation of the channel at less depolarizing membrane potentials [87]. Interestingly, the same authors also report *de novo* germline mutations in two children with a previously unrecognized syndrome of primary aldosteronism and neuromuscular abnormalities [87]. In addition, up-regulation of *CACNA1D* expression has been linked to prostate cancer progression [88]. Specifically, *CACNA1D* up-regulation and concomitant increases in Ca_V_1.3 protein levels were identified in tissue biopsies from subjects with malignant prostate cancer compared with non-malignant controls [88]. Ca_V_1.3 functional involvement in LNCaP cells was shown by both pharmacologic blockade of the channel and genetic approaches knocking down *CACNA1D* expression, both of which resulted in a decrease in androgen-stimulated Ca^++^, and suppressed the associated androgen receptor transactivation and cell growth [88]. Knock-down of the *CACNA1C*-encoded Ca_V_1.2 channel did not show any effects [88]. Additional evidence for a pathologic Ca_V_1.3 up-regulation is based on pharmacologic studies with the cyclooxygenase-2 inhibitor, celecoxib, which exerts chemopreventive effects against multiple cancers, including prostate cancer [89]. These effects appear to be mediated at least in part by down-regulation of androgen receptors [89]. However, it is tempting to speculate that down-regulation is the result of celecoxib’s inhibitory effects on Ca_V_1 channel-mediated L-type Ca^++^ currents [89,90].

Interestingly, the Ca_V_1 blocker, nifedipine, has been implicated in the proliferation of certain cancers, however, its effects are likely not mediated by blocking Ca_V_ channels [91]. One recent study demonstrated that nifedipine promoted both the proliferation and migration of breast cancer *in vitro* and *in vivo* [91], while another Ca_V_1 blocker, verapamil, did not affect proliferation [91]. Similarly, the application of nifedipine in the presence of depolarizing KCl concentrations did not reduce the intracellular Ca^++^ concentration, suggesting a Ca_V_-independent mechanism [91]. Rather, a mechanism involving down-regulation of miRNA524-5p has been proposed based on *in vitro* studies in MDA-MB-231 cells [91].

### 3.2. Ca_V_3 Channels

One of the earliest studies implicating low voltage-activated Ca_V_3 channels in cancer progression was derived from studies testing the sensitivity of Ca^++^ entry pathways to Ni^++^ in non-excitable cells. Synthesis and testing of novel compounds resulted in the inhibition of Ca^++^ influx and the concomitant stereoselective inhibition of cell proliferation in cancer cells expressing Ca_V_3.2, but not those devoid of Ca_V_3.2 expression [92]. Specificity for the Ca_V_3.2 subtype was demonstrated by inhibition of proliferation of HEK293 cells transfected with the *CACNA1H* gene encoding Cav3.2 [92].

Interestingly, the opposite was found in MCF-7, a human breast adenocarcinoma cell line. While overexpression of full-length Ca_V_3.1 suppressed cell proliferation, knockdown of the *CACNA1G* gene encoding for Cav3.1 promoted cell proliferation [93]. In contrast, Ca_V_3.2 overexpression did not affect cell proliferation [93]. In their study, the authors showed convincing evidence of a differential distribution of Ca_V_3.1 and Ca_V_3.2 channels at plasma membranes of apoptotic and non-apoptotic cells, respectively.

Similarly, Ca_V_3.1 channel expression was present in human laryngeal squamous cell carcinoma tissue and cell lines [94]. Notably, *CACNA1G* gene knock-down and the Ca_V_3 channel blocker, mibefradil, significantly reduced the rate of cell proliferation in this cancer [94].

However, due to the lack of Ca_V_3 subtype specific pharmacologic blockers and/or agonists, many studies have investigated the group of Ca_V_3 channels as a whole. For instance, in a comprehensive study by investigating the expression profile of Ca_V_ channels in human melanocytes and a number of melanoma cell lines and biopsies. It was observed that, Ca_V_3 channels were selectively expressed in melanoma [95]. More importantly, mildly hypoxic conditions reminiscent of the hypoxic environment of skin and the high oxygen demand in metastasis increased Ca_V_3 expression in melanoma [95], suggesting a role for CaV3 channels in proliferation of malignancies. The same authors reported also that clinically relevant Ca_V_3 blockers, mibefradil and pimozide, exerted dual effects on cell viability, specifically a reduction of cell viability and initiation of apoptosis [96]. Notably, a temporal analysis of intracellular signaling events revealed that endoplasmic reticulum stress and inhibition of basal macroautophagy precede activation of apoptotic pathways in both M16 and JG melanoma cells, suggesting subtype-specific mechanisms.

Ca_V_3 channel overexpression was also detected in glioblastoma [97]. Pharmacological inhibition and small interfering RNA-mediated Ca_V_3 channel knockdown resulted in reduced viability and clonogenic potential, as well as the induction of apoptosis [97]. Ca_V_3 blockers effectively reduced phosphorylation of Akt, Rictor and anti-apoptotic proteins in glioblastoma cells, and induced activation of caspases [97]; in contrast, genetic knock-down of Ca_V_1 channels had no effect on cellular proliferation [97].

The same mechanism is likely targeted by endostatin, which has been shown to selectively inhibit Ca_V_3.1 and Ca_V_3.2, but not Ca_V_3.3 or Ca_V_1.2 channels recombinantly expressed in human embryonic kidney (HEK) or Chinese hamster ovary (CHO) cells [98]. Furthermore, endostatin dose-dependently reduced cell proliferation and migration of glioblastoma U87 cells [98].

In prostate cancer, exposure of LNCaP prostate cancer cells to sodium butyrate resulted in morphological and molecular differentiation, including a significant up-regulation of Ca_V_3.2 channels [99]. Furthermore, sodium butyrate yielded detectable fast-inactivating, Ni^++^-sensitive Ca^++^ currents, which enhanced cellular proliferation [99].

### 3.3. Auxiliary Ca_V_-α_2_δ Subunits

The pore-forming Ca_V_-α_1_ subunit of high voltage-activated Ca_V_ channels is associated with auxiliary subunits, while low voltage-activated Ca_V_ channels do not co-assemble with auxiliary subunits [100]. Auxiliary Ca_V_ subunits include the groups of Ca_V_-β subunits (β_1_–β_4_), Ca_V_-α_2_δ subunits (α_2_δ_1_^−^ α_2_δ_4_), and Ca_V_-γ subunits (γ_1_-γ_4_) and contribute to the functional diversity of the high voltage-activated Ca_V_ channels [100].

The *CACNA2D2* gene encoding the Ca_V_-α_2_δ_2_ subunit is located on chromosome 3p21, a well-defined tumor suppressor locus [101]. Early after the genetic identification and functional analysis of the Ca_V_-α_2_δ_2_ subunit [102], single nucleotide polymorphisms associated with small cell lung cancer were detected in the *CACNA2D2* gene [103]. Similarly, there was an observed 50% loss of expression in cancer cell lines [104].

Subsequent experiments utilizing adenoviral vector-mediated gene transfer to overexpress the Ca_V_-α_2_δ_2_ subunit in non-small cell lung cancer revealed a significant inhibition of tumor growth [105], mediated by increased apoptosis and the preceding disruption of mitochondrial membrane integrity [105].

In contrast, more recent studies on prostate cancer have shown that overexpression of the Ca_V_-α_2_δ_2_ subunit leads to increases in the rate of cell proliferation *in vitro*, and that xenografts of LNCaP cells overexpressing the Ca_V_-α_2_δ_2_ subunit into nude mice are more tumorigenic, showing evidence of increased cellular proliferation and angiogenesis [106]. In support of the direct mechanistic role of the Ca_V_-α_2_δ_2_ subunit in their model, the authors were able to show efficacy of the selective Ca_V_-α_2_δ_2_ subunit blocker, gabapentin, in reducing tumors in the xenografts [106].

Similarly, the related *CACNA2D3* locus, encoding the Ca_V_-α_2_δ_3_ subunit, has been identified as a frequent site of methylation in gastric cancer [107]. Furthermore, methylation of *CACNA2D3*, but not *CACNA2D2* was associated with significantly shorter survival [107], making it a useful prognostic marker for patients with gastric cancer. The tumor repressive properties of the Ca_V_-α_2_δ_3_ subunit were also observed in *in vitro* models, where overexpression resulted in reduced cell growth and adhesion, while down-regulation using small interference RNAs had the inverse effect [107]. Methylation-dependent silencing of *CACNA2D3* was proposed as a biomarker for the risk of development of metastatic disease in breast cancer, where methylation correlated strongly with visceral and metastatic disease independent of α-estrogen receptor expression [108].

More recently, similar tumor suppressor function of *CACNA2D3* was identified for the development and progression of nasopharyngeal carcinoma [109] and esophageal squamous cell carcinoma [110].

### 3.4. Mechanistic Considerations

While there is clear evidence that supports an involvement of Ca_V_ channels and auxiliary Ca_V_ subunits in the proliferation of cancer cells, several questions remain regarding the underlying mechanism. A new hypothesis involves the formation of complexes comprised of Ca^++^ activated potassium (KCa) and Ca_V_ channels for review, see [86]. For example, large-conductance BKCa channels expressed in prostate cancer LNCaP cells set the resting membrane potential at approximately −40 mV, thereby promoting constitutive Ca^++^ entry through Ca_V_3.2 channels [111]. Furthermore, both channels form macromolecular complexes, where Ca_V_3 channels are located in clusters of BK channels [111]. This co-localization thus provides for an effective feed-forward loop of constitutive Ca^++^ entry, which in turn regulates the membrane potential by activating BKCa channels [86].

Further studies are needed to investigate the biophysical properties of Ca_V_ channels, particularly their association with other ion channels in macromolecular complexes as well as their association with auxiliary subunits.

## 4. Voltage-Gated Na^+^ Channels

Voltage-gated Na^+^ channels selectively allow the passage of Na^+^ ions into the cells resulting in membrane depolarization and play a pivotal role in initiation and conduction of action potential in excitable cells including neurons, heart and skeletal tissue [112]. They are distinguished into nine families identified thus far (Nav1.1–1.9) based on the alpha subunit which is the essential pore forming unit that facilitates Na^+^ transport across the membrane. The alpha subunits are found to associate with one or more beta subunits (ᵝ1^−^ ᵝ4). While the alpha subunits are functional by themselves, the beta subunits can regulate the expression and gating of Nav channels. In recent years, the increase in expression of Nav channels in a broad number of cancers has been extensively reported [113,114]. Cancers seem to express variable and multiple Nav channels, with certain types expressing a predominant alpha subunit. For instance, Nav1.5 is most abundantly expressed in breast and colon cancer cell lines [115] while Nav1.6 in cervical cancer and Nav1.7 in prostate cancer [113,114]. Furthermore, some cancers express alternatively spliced more active, neonatal isoforms, such as the neonatal Nav1.5, Nav1.7 and Nav1.9 [116]. Nav expression and activity is greatly enhanced in strongly metastatic cell lines such as the MDA-MB-231 breast cancer cell line in comparison to weakly metastatic MCF 7 cell lines [117]. Similar observation is consistent in prostate cancer cell lines, wherein strongly metastatic PC3 cells demonstrate significantly higher expression of Nav1.9 alpha subunit in comparison to the weakly metastatic LNCap cell line [118,119]. These observations have been corroborated *in vivo* and as well as in human cancer biopsies [120]. However, few studies suggest that inhibiting Nav channel activity reduces cell proliferation while the vast majority of the data appear to associate Nav channel activity to metastasis, invasion, galvanotaxis, endocytosis and secretion, as these effects are effectively inhibited by tetrotodoxin (TTX) a selective Nav channel inhibitor and several other Nav channel blockers while having no effect on cell proliferation [121,122,123,124]. Given the direct correlation of Nav channel expression to the metastatic potential of tumors and the ability of of Nav inhibitors to block metastasis, Nav channels have emerged as novel biomarkers and as well as therapeutic targets for certain cancers.

## 5. Voltage-Gated Chloride Ion Channels

Chloride ion channels are a diverse group of membrane proteins that facilitate the transport of chloride ions across cell membrane and intracellular organelles. They play a pivotal role in a number of physiologically important functions ranging from trans-epithelial secretion, pH regulation, cell volume regulation, proliferation and membrane potential stabilization [125]. Depending on their mode of activation, Cl^−^ channels are classified as voltage dependent (CLC family), cAMP regulated (CFTR), or calcium dependent chloride ion channels. The transport of chloride is also regulated by volume-sensitive organic osmolyte/Anion channels (VSOAC), which regulate the flow of organic solutes and anions in response to changes in cell volume. The voltage dependent CLC family of ion channels was initially identified in torpedo membranes (CLC-0) followed by nine different CLC proteins with similar sequence homologies being identified thus far in mammalian systems including CLC 1-7, CLCka, and CLCkb. In recent years, CLC family of Cl^−^ channels have been implicated in a number of cancers. Gliomas, in particular, seem to express higher levels and activities of CLC 2 and CLC 3 with enhanced ability to efflux Cl^−^, thus contributing to depolarized membranes [126]. Cl^−^ channel blockers or siRNA mediated knockdown of CLC 3 have been shown to reduce Cl^−^ currents and inhibit proliferation of gliomas. The inhibitory effect following CLC 3 block on glioma proliferation has been mainly attributed to its regulatory effect on cell volume control during the cell cycle [127,128].

Cell proliferation comprises an accurate control of cell volume that is achieved by the fine government of osmolarity. Therefore, changes in intra/extra-cellular ionic concentration upon activation of ion channels can directly alter cell volume and, therefore, provide a contribution to cell proliferation process. Water molecules follow the flux of ions through ion channels creating changes in cell volume, cytoplasm, and condensation/shrinkage described as pre-mitotic condensation (PMC) prior to mitosis [73,129]. Impairment in PMC can result in delay or potentiation of the cell cycle. PMC or cell shrinkage is primarily attributed to the loss of fluids associated with Cl^−^/K^+^ ion efflux. It has been observed that Cl^−^ conductance is increased in the M phase, CLC 3 channel is enriched in the PMC phase, furthermore inhibiting Cl^−^ channel activity or knockdown of CLC 3 results in lengthened cell cycles. Similarly, CLC 3 expression, activity and its possible role in cell volume control has been implicated in a number of cancers including nasopharyngeal carcinoma, endometrial and ovarian cancer cells [130,131,132]. While the role of voltage-gated *CiLC* 3 in cell volume regulation is still under debate, nevertheless, the enhanced expression of CLC family proteins in certain cancers and alleviation of the progression of these cancers by inhibiting Cl^−^ currents, offers a potential role for Cl^−^ ion channels in cancer and merits further investigation.

## 6. VGICs as Therapeutic Targets for Cancer

Given the enhanced expression and activity of various voltage-gated ion channels in cancer and their role in key events contributing to initiation and progression of the disease, VGICs have emerged as a novel therapeutic target for treating cancers. A number of preclinical *in vitro* and *in vivo* studies targeting VIGC have led to valuable insights on the usefulness and limitations of existing approaches, while fostering the need for further strategies to effectively target ion channels such that, its role in cancer can be harnessed to its complete potential. Several small molecules targeting VGICs that are/were clinically relevant for other non-cancer conditions incidentally, which also are VGIC channel blockers, have been investigated in preclinical cancer studies for their therapeutic potential. For example, imipramine, an antidepressant known to block Kv10.1, inhibits proliferation of cancer cells including ovarian, osteosarcoma and small cell lung carcinoma by apoptosis. However, in melanoma cell lines, it inhibits proliferation without affecting apoptosis [133,134,135,136,137,138]. Astemizole, an anti-histamine, is able to inhibit both Kv10.1 and Kv11.1 channels, contributing to inhibition of cancer cell proliferation *in vitro* and *in vivo* [139,140,141]. Several antiarrhythmic agents of the class III antiarrhythmics are known to block Kv11.1 channels. Anti-arrhythmic agents Way 123,398 and E4031 are selective blockers of Kv11.1 have been shown to impart therapeutic benefit in cancer as observed in various *in vitro* and pre-clinical models of cancer [135,142,143]. A major limitation that prevents the use of the Kv10.1 and Kv11.1 blockers in cancer is their adverse effects on cardiac physiology, resulting in prolonged QT intervals, which may further lead to fatal ventricular arrhythmias [144].

Thus a number of diligent studies are focused on understanding the structure and gating of Kv11.1 channels with the objective of identifying molecules that can circumvent the adverse effect of prolonged QT intervals while still retaining the Kv11.1 channel inhibition. For example, roscovitine, a cyclin dependent kinase inhibitor currently in phase II trials for treating cancers, is able to inhibit kv11.1 in clinically relevant doses [145,146,147] and yet, no adverse effects of arrhythmia have been reported thus far. The lack of arrhythmia by roscovitine has been attributed to its specific, rapid reversible binding kinetics to only open channel conformation of Kv11.1 and a lack of in use dependence commonly observed with potent Kv11.1 channel blockers. Similarly, verapamil, a voltage dependent Ca^++^ blocker which also inhibits kv11.1, fails to elicit a prolonged QT interval in subjects, probably due to its masking effect of Kv11.1 inhibition with that of voltage^−^ dependent Ca^++^ channel inhibition and lack of action potential dependence [148,149]. Furthermore, peptide toxins, obtained from various animal venoms including, snakes, scorpions, sea anemones are potent Kv channel inhibitors, have served as a critical pharmacological tools in understanding the mechanisms leading to Kv channel inhibition and identifying toxins and designing small molecules selective for a specific Kv channel [150]. Unlike small molecules, which bind to the inner channel pore, Kv channel peptide toxins bind to the outer channel pore and occlude it or act as gating modifiers by binding to the voltage sensor domain of the Kv channel, leading to enhanced stability of the closed state and resulting in a channel that is more difficult to open. Studies focused on peptide toxins have led to identification and designing of peptides derived from toxins with enhanced specificity for a particular Kv channel. For instance, anemone toxin APETx1 is a gating modifier with high specificity for Kv11.1 [151,152]. Similarly, a modified sea anemone toxin-ShK-186/SL5 specifically targets Kv1.3 expressed in auto-reactive memory/effector T cells implicated in autoimmune disease and ameliorates the disease symptoms in animal models [153]. While upregulation Ca_V_1.3 has been observed in prostate cancer and its pharmacological blockade inhibits growth in prostate cancer cell lines, the Ca_V_1 blocker, nifedipine, however, has been implicated in the proliferation of certain cancers and its effects are likely not mediated by blocking Ca_V_ channels [88,91]. One recent study demonstrated that while nifedipine promoted both the proliferation and migration of breast cancer *in vitro* and *in vivo* [91], verapamil did not affect proliferation [91]. The Cav3 inhibitors including mibefradil are able to inhibit cancer cell proliferation [94,96].

Several FDA–approved VGSC blockers that are currently used as anticonvulsants, antiarrhythmic, or tricyclic antidepressants [154,155] could be potentially repurposed in cancer therapy. For example, phenytoin, an anti-convulsing drug, effectively blocks VGSC and does not only inhibit the migration and invasion of metastatic MDA-MB-231 cells *in vitro* but also in clinically relevant doses is able to significantly reduce tumor growth, cancer cell proliferation *in vivo*, invasion and metastasis [115,156,157].

Novel hydroxyamide compounds developed using rational drug designing approaches, based on the binding site of phenytoin to VGSCs, have demonstrated significant inhibition of voltage-gated sodium channel activities and increased inhibition of androgen–independent prostate cancer cell growth *in vitro* in comparison to phenytoin and significant ability to reduce prostate cancer *in vivo* as well [124,158]. Moreover, it has been observed that the beneficial effects of EPA in inhibiting prostate cancer cell proliferation and metastasis seems to be mediated by its inhibition of voltage-gated sodium channels.

Lack of specific inhibitors has been a major limitation in targeting voltage-gated chloride channels in cancer therapy. Several known anticancer molecules including tamoxifen, 5-nitro-2-(3-phenylpropyl-amino) benzoic acid (NPPB) that are able to inhibit chloride channel currents [131,159] also indeed inhibit cancer cell proliferation.

Chlorotoxin, a 36 amino acid peptide derived from scorpion venom, is able to inhibit glioma progression [160]. Cholortoxin and its derivatives are known to bind specifically to CLC 3 channels and inhibit the channel activity, which in part explains its inhibitory effect on gliomas, as CLC 3 is known to play a critical role in glioma proliferation and progression. However, cholorotoxin, also binds to matrix metalloproteinase-2 (MMP2) [161] resulting in reduced membrane-associated MMP2 activity and therefore inhibiting migration of glioma cells. Incidentally, CLC 3 and MMP2 are both over and co-expressed specifically in gliomas rather than in the surrounding normal tissue. Thus, chlorotoxin has emerged as not only a therapeutic agent but also a diagnostic agent for glioma therapy. TM-601, an I^131^ synthetic derivative of chlorotoxin, has been approved by the FDA for glioma therapy and diagnostics [162,163].

A more selective targeting of a specific channel has been achieved by generating antibodies against it. Antibodies serve as an extremely specific molecule for inhibiting a peptide/protein target. Selective monoclonal antibodies have been generated that are highly specific for Kv10.1 and that are not able to cross the blood brain barrier. These antibodies are known to impart moderate anti-tumor effects and can also be used as diagnostic or drug delivery agents to deliver cytotoxic drugs selectively to the tumor expressing Kv10.1 channels [36].

Selective inhibition of ion channels have also been possible with the use of molecular tools such siRNAs. For instance selective knockdown of Kv10.1 with siRNA reduces Kv10.1 expression and cell proliferation in several cancer cell lines [39]. Similarly, plasmid mediated expression of shRNA-herg1 and shRNA-herg1/1b inhibited the cellular growth rate, viability and colony formation of SH-SY5Y neurobalstoma cells [164]. Further, in the same study, it was determined that cells intra-tumor injection of shRNA-hERG1/1b inhibited the growth of SH-SY5Y tumors inoculated subcutaneously in nude mice. Similarly, CLC 3 specific siRNAs are able to inhibit CLC 3 mediated proliferation in cancer cell lines [165,166]. The voltage gated channel blockers contribute inhibition in cell proliferation either due to cell cycle arrest or apoptosis via caspase 3 activation. The cell cycle arrest is observed either at the G0/G1 phase or the G2/M phase through the accumulation of tumor suppressors p27kip1 and p21CIP1 and down regulation of cyclins or cyclin dependents kinases critical for cell cycle progression [167]. The G2/M arrest has been attributed to the regulatory effect of VGIC in cell volume changes during cell cycle progression [168].

### VGIC Activators for Cancer Therapy

More recently, it has been observed that ion channel activators have also proven beneficial in inhibiting cancer cell proliferation. For example, we have observed that pharmacologic stimulation of Kv11.1 with the selective activator NS1643 in various breast cancer cells results in marked decrease in cell proliferation by activation of a senescent-like cellular program that is characterized by a cell cycle arrest at G1 phase, decreased levels of tumor markers such as cyclin E2 and increased level of tumor suppressors such as p21^Waf1/Cip1^ and p16^INK4A^ [48,169]. This dramatic inhibitory effect of NS1643 is mediated by Ca^++^ driven signaling mechanism that contributes to selective degradation of cyclin E2 [48] and enhanced expression of p21^Waf1/Cip1^ [170], Moreover, Kv11.1 openers have shown to exhibit less cardiovascular adverse effects compared to Kv11.1 blockers. Similarly, it has been observed that bufalin, a bufadienolide isolated from toad venom, is a novel CLC-3 activator that has anti-tumor activity and contributes to cancer cell apoptosis through inhibition of the PI3K/Akt/mTOR pathway [171]. This suggests that stimulation of VGIC could be a valid alternative or additive therapeutic approach to treat cancer. We have summarized the potential therapeutically approaches for individual VGIC in Table 1.

**Table 1:** Voltage-gated ion channel in cancer cell proliferation.

VGIC	Expression	Therapeutic Approach	Effect	Ref
Voltage gated K^+^ channel Kv 10.1 Kv 11.1	Cervical cancer, Breast cancer Ovarian Cancer Osteosarcoma Glioma Melanoma small cell Lung cancer Myeloid leukemia	Small molecule channel inhibitors Kv10.1; Astemizole, Imipramine Kv11.1; Way123398, E4031 Small molecule channel activators NS1643 Antibody Kv10.1 Toxins Kv11.1; Ergotoxin siRNA/shRNA Kv10.1, Kv11.1	Reduced proliferation by increasing apoptosis or cell cycle arrest at G0/G1, G1/S or G2/M phase	[36,39,48,133,143,151,152,164,169]
Voltage gated Ca^++^ channel Cav 1/Cav 1.3 Cav 3/Cav 3.1	Adrenal Adenomas Prostate cancer Melanoma Glioblastoma	Small molecule Inhibitors Cav; mibefradil, TTL1177, endostatin siRNA/shRNA Cav3.1, Cav1.3	Reduced cell proliferation by inducing apoptosis	[88,94,95]
Voltage gated Na^+^ channel Nav 1.5, 1.6, 1.7, 1.9	Prostate cancer Ovarian cancer Cervical cancer Breast cancer	Small molecule Inhibitors Phenytoin, novel hydroxyl amide Toxins Tetradotoxin	Reduced cell proliferation via cell cycle arrest observed with the small molecule inhibitors.	[156,157,158]
Voltage gated Cl^−^ channel	Gliomas	Small molecule inhibitors 5-nitro-2-(3-phenylpropyl-amino) benzoic acid (NPPB)NPPB Small molecule activators Bufadienolides Toxin Chlorotoxin siRNA CLC-3	Reduced proliferation by cell cycle inhibition at G1, G2/M phase via cell volume regulation Channel activators induce apoptosis	[131,159,160,161,162,163,164,165,166,171]

## 7. Conclusions

Voltage-gated ion channel activity is an important event that controls several cellular functions including generation, maintenance of action potentials, and secretion. The role of VGICs in cancers is being extensively investigated and has led to valuable insight into mechanisms by which VIGCs contribute to tumorogenesis. In light of current literature and the availability of well characterized VGIC inhibitors/activators already in market, a compelling opportunity for further advancing the studies that characterize the role of VIGC in cancer and targeting them as therapeutics for cancer has been provided.
